# Effects of Ankle Arthrodesis on Biomechanical Performance of the Entire Foot

**DOI:** 10.1371/journal.pone.0134340

**Published:** 2015-07-29

**Authors:** Yan Wang, Zengyong Li, Duo Wai-Chi Wong, Ming Zhang

**Affiliations:** 1 Interdisciplinary Division of Biomedical Engineering, Faculty of Engineering, The Hong Kong Polytechnic University Hong Kong, China; 2 The Hong Kong Polytechnic University Shenzhen Research Institute, Shenzhen, China; 3 Key Laboratory of High Efficiency and Clean Mechanical Manufacture, School of Mechanical Engineering, Shandong University, Jinan, China; Queen Mary University of London, UNITED KINGDOM

## Abstract

**Background/Methodology:**

Ankle arthrodesis is one popular surgical treatment for ankle arthritis, chronic instability, and degenerative deformity. However, complications such as foot pain, joint arthritis, and bone fracture may cause patients to suffer other problems. Understanding the internal biomechanics of the foot is critical for assessing the effectiveness of ankle arthrodesis and provides a baseline for the surgical plan. This study aimed to understand the biomechanical effects of ankle arthrodesis on the entire foot and ankle using finite element analyses. A three-dimensional finite element model of the foot and ankle, involving 28 bones, 103 ligaments, the plantar fascia, major muscle groups, and encapsulated soft tissue, was developed and validated. The biomechanical performances of a normal foot and a foot with ankle arthrodesis were compared at three gait instants, first-peak, mid-stance, and second-peak.

**Principal Findings/Conclusions:**

Changes in plantar pressure distribution, joint contact pressure and forces, von Mises stress on bone and foot deformation were predicted. Compared with those in the normal foot, the peak plantar pressure was increased and the center of pressure moved anteriorly in the foot with ankle arthrodesis. The talonavicular joint and joints of the first to third rays in the hind- and mid-foot bore the majority of the loading and sustained substantially increased loading after ankle arthrodesis. An average contact pressure of 2.14 MPa was predicted at the talonavicular joint after surgery and the maximum variation was shown to be 80% in joints of the first ray. The contact force and pressure of the subtalar joint decreased after surgery, indicating that arthritis at this joint was not necessarily a consequence of ankle arthrodesis but rather a progression of pre-existing degenerative changes. Von Mises stress in the second and third metatarsal bones at the second-peak instant increased to 52 MPa and 34 MPa, respectively, after surgery. These variations can provide indications for outcome assessment of ankle arthrodesis surgery.

## Introduction

A growing population with ankle arthritis [1,2] has led to the imperative need for effective ankle reconstruction surgeries. Ankle arthrodesis, which accounts for more than 85% of ankle surgeries [3], has been reported to be an effective surgery for pain relief and retaining plantigrade foot function [4]. However, postoperative complications of ankle fusion, such as adjacent joint degeneration, foot pain, and limited foot motion, are common [4–9]. A high prevalence of ipsilateral hind- and mid-foot arthritis associated with deterioration has been reported in retrospective clinical studies [6,9–11].

Surgical interventions change the biomechanical behavior of the foot due to the interdependent interactions between its structures. Sufficient understanding of the biomechanical effects of ankle arthrodesis on the entire foot and ankle is critically important to ensure improvement following surgery.

Biomechanical studies have been undertaken to understand the consequences of ankle arthrodesis. Gait analysis has shown that a foot with ankle arthrodesis reduces walking speed compared to a normal foot due to a reduction in cadence and stride length [4,9,12–15], whereas the time proportion of the stance phase barely changes. Multi-body models of the musculoskeletal system have been developed to estimate the biomechanical information relating to the muscle and joint forces and relative motions among segments, but detailed biomechanical behavior in individual bones [16–18] and their articulating joints has not been provided.

Cadaveric experiments have allowed the assessment of the movements of individual segments [19–22] and the contact pressure at the interfaces of some articulations [23]. These measurements implicate potential outcomes and complications of foot surgeries [24–27]. However more detailed biomechanical information, such as stress distributions within bones and soft tissues is not easy to measure due to a lack of measurement techniques. It is also difficult to represent the muscle activities during gait with cadaveric measurements [17].

Considering the limitations of experimental studies, computational approaches such as finite element analysis can be a complementary tool to enhance the biomechanical understanding of human musculoskeletal structures. Simulations can provide insight into biomechanical parameters, such as the stress/strain distribution in each component, contact pressure at articular interfaces, and deformation and relative movement. Several simplified finite element models, either with partial foot geometries or two-dimensional models, have been developed to evaluate ankle arthrodesis surgery and to assist in surgical planning [28–35]. These investigations have provided valuable insight into surgical consequence generally around the revised regions. Three-dimensional finite element models with more detailed representations of the major anatomical structures are capable to understand the sophisticated interactions among the entire foot segments [36–40].

The aim of this study was to explore the biomechanical response of the entire foot to ankle arthrodesis using a comprehensive finite element model of the foot and ankle. This exploration would be beneficial to identify the risk factors associated with surgical failures and to optimize surgery protocols.

## Methods

### Ethics Statement

Ethical approval for cadaveric experiment and human motion analysis was granted by The Hong Kong Polytechnic University Human Subject Ethics Committee (reference number HSEARS20070115001-01). The subject participated in the gait experiment was informed of the experimental procedures and gave written informed consent for participating in the magnetic resonance imaging scanning and gait measurements and for publishing these case details.

### Model development

**Fig 1:**
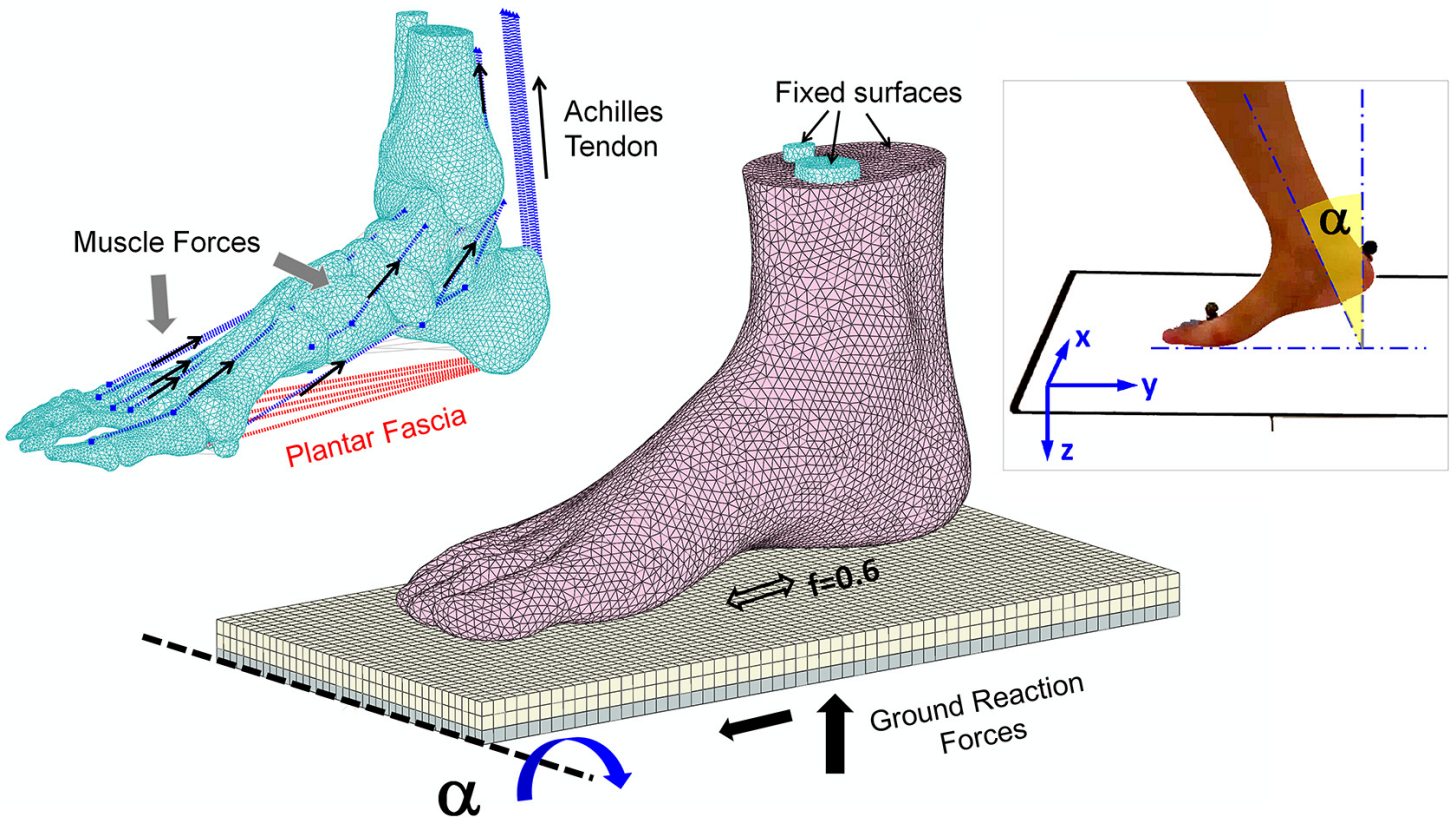
The three-dimensional finite element model of the foot and ankle and application of boundary and loading conditions.

A three-dimensional finite element model of a female foot and ankle consisting of 28 bony segments, 103 ligaments, plantar fascia, major muscle groups, and the bulk of encapsulated soft tissue was previously developed [40,41]. The geometries of the bony structures and the encapsulated soft tissue were reconstructed from magnetic resonance images with 256×256 pixels (resolution 0.625 mm) of 2mm intervals from the right foot (size of 38) of a normal adult (Height 164 cm; Weight 54 kg), who did not have any pathologies or injuries in lower limbs, using MIMICS (Materialise, Leuven, Belgium). The geometric models were assembled and meshed (Fig 1) using the ABAQUS finite element package (Dassault Systèms Simulia Corp., Providence, RI, USA). For simplification, the articular cartilages are not separated from the corresponding bone surfaces in the segmentation process. The articulation behaviors of the major joints were simulated as frictionless surface-to-surface contacts, and non-linear contact stiffness [42] was assigned to represent the cartilaginous layer. The phalanges of the four lesser digitals were connected together using 2 mm thick of cartilage structures. The 103 ligaments and the plantar fascia were simulated as wire features through connected insertion points on corresponding bones and represented as tension-only truss elements. Nine muscle groups—the tibialis anterior, extensor digitorum longus, extensor hallucis longus, peroneus longus, peroneus brevis, Achilles tendon (merged triceps surae), tibialis posterior, flexor hallucis longus, and flexor digitorum longus—were represented as axial connector elements, connecting the attachment points of the muscles to the bones and allowing the application of muscle forces.

The ground support was simulated as two layers of plates tied together and meshed into hexahedral elements. The upper layer was assigned with an elastic property to simulate the concrete ground support and the lower layer was set as a rigid body for the application of boundary and loading conditions. The interaction between the foot plantar surface and the support surface was simulated as contact with friction. The coefficient of friction was set to 0.6 [43].

The bones, ligaments, cartilages, and ground plate were simplified as homogeneous, isotropic, and linearly elastic materials. The two material constants of Young’s modulus E and Poisson’s ratio ν were given to describe the elasticity. The two constants of the bones [44], cartilage [42], ligaments [45], and plantar fascia [46] were obtained from literature sources. The encapsulated soft tissue and the skin were set as nonlinear hyperelastic materials. The hyperelastic behavior of the soft tissue was described using a second-order polynomial strain energy potential expression (Eq 1) (ABAQUS 6.4 User’s Manual) in the form of

$$U=\sum_{i+j=1}^{2}C_{ij}{\left({\bar{I}}_{1}-3\right)}^{i}{\left({\bar{I}}_{2}-3\right)}^{j}+\sum_{i=1}^{2}\frac{1}{D_{i}}{\left(J_{el}-1\right)}^{2i}$$ (Eq. 1)

where *U* is the strain energy per unit of reference volume; ${\bar{I}}_{1}$ and ${\bar{I}}_{2}$ represent the first and second deviatoric strain invariants; and *C* and *D* are the input coefficients of hyperelasticity parameters. The constitutive constants *C* and *D* were determined by a stress-strain curve obtained from experiments [47]. The properties of the skin was represented using the Ogden strain energy potential formulation [48]

$$U=\frac{2\mu }{\alpha^{2}}\left(\lambda_{1}^{\alpha }+\lambda_{2}^{\alpha }+\lambda_{3}^{\alpha }-3\right)$$ (Eq. 2)

where *λ*_1−3_ are the deviatoric principal stretches, and coefficient describing particular material was set as *α* 18 and *μ* 0.122 [49]. The plantar fascia and ligaments were meshed into the truss elements, while other tissues including the bones, the cartilage and the encapsulated soft tissue were meshed into linear tetrahedral elements. The mesh and material properties are shown in Table 1.The ankle arthrodesis model was simulated by fusing the talus and tibia bones together based on the normal foot model. In a real ankle arthrodesis surgery, the ankle joint is fused using screws, plates, or pins to constrain the joint motion generally in the 5- to 10-degree valgus position. Based on debates that fusing the ankle in a neutral position allows the use of any remaining mid-foot motion, which could compensate for some ankle joint motion and give better functional results [4,12,50–53], the contact pair between the talus and tibia bones was tied together in a neutral position to represent the surgical intervention in the finite element simulation.

**Table 1:** Material property and mesh element type for the foot model components.

Component	Element Type	Young’s Modulus *E* (MPa)			Poisson’s Ratio *v*		Cross-section Area (mm^2^)	
**Bone[44,79]**	4-node linear tetrahedron	7300			0.3		-	
**Cartilage[42]**	4-node linear tetrahedron	1			0.4		-	
**Ligaments[45]**	2-node linear 3-D truss	260			-		18.4	
**Plantar Fascia[46]**	2-node linear 3-D truss	350			-		58.6	
**Ground**	8-node linear brick	17000			0.1		-`	
**Skin[48,49]**	3-node triangular shell	*α* 18			*μ* 0.122			
**Encapsulated Soft Tissue[47]**	4-node linear tetrahedron	*C*_10_ 0.08556	*C*_01_-0.05841	*C*_20_ 0.03900	*C*_11_-0.02319	*C*_02_ 0.00851	*D*_1_ 3.65273	*D*_2_ 0.00000

### Boundary and Loading Conditions

**Fig 2:**
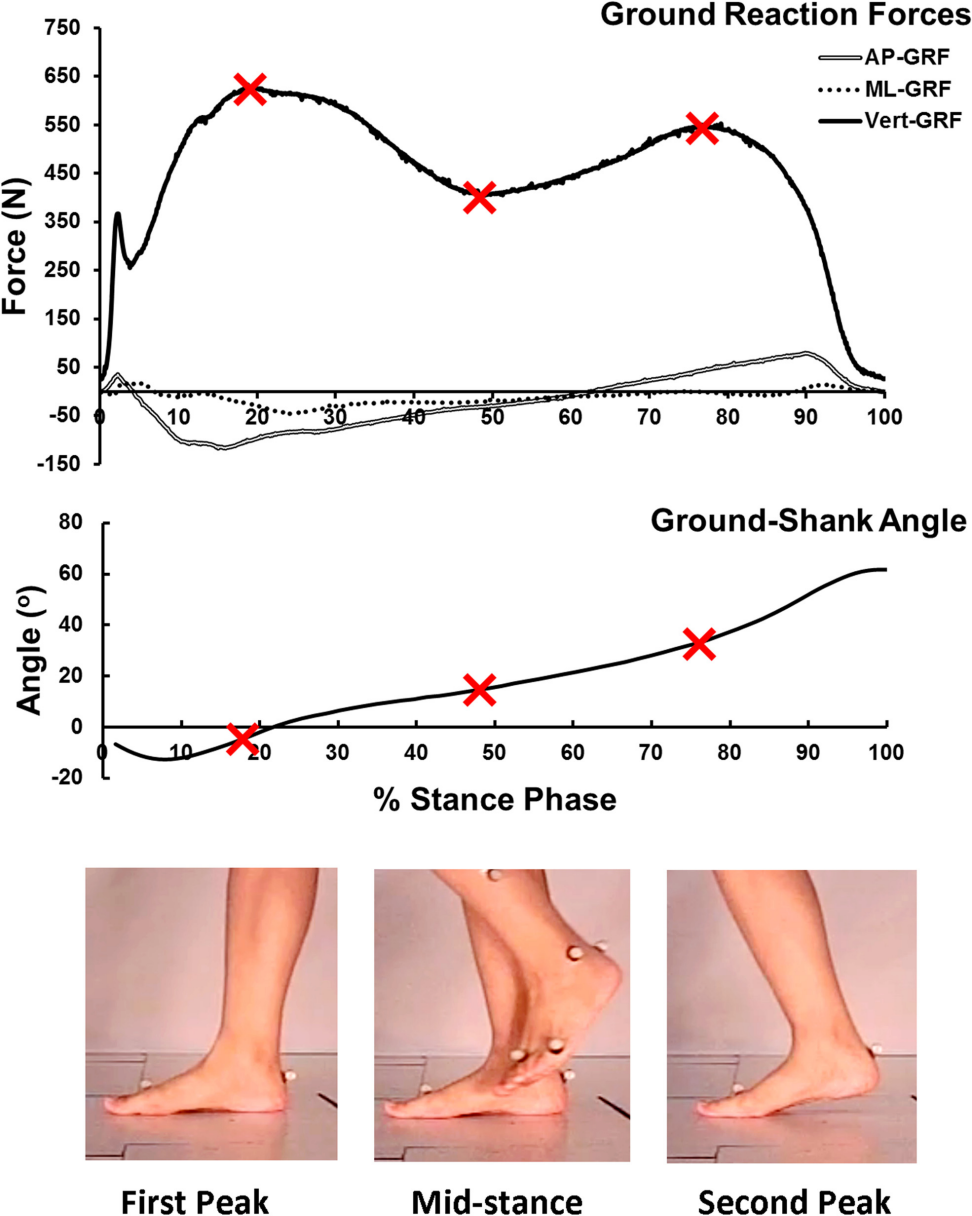
Ground reaction forces and ground-shank angle recorded in the gait analysis and the three instants, first-peak, mid-stance, and second-peak, for simulation. The three instants were marked in the curves.

Boundary conditions and ground reaction forces were derived from gait analysis, which was carried out using the Vicon Motion Analysis System (Vicon, Oxford Metrics, Oxford, UK) from the same subject as the foot model. Sixteen retro-reflective markers were attached to the subject’s lower limbs to define seven segments, the pelvis, two thighs, two lower legs, and two feet. Static calibration was conducted when the subject stood on an AMTI force platform (Advanced Mechanical Technology, Inc., Watertown, MA, USA). The position of the right foot shank was recorded by creating an angle between the tibia bone and the global system, depicted as angle α in Fig 1. The angle in the sagittal plane was calculated. The curve is shown in Fig 2. Ground reaction forces in the vertical, anteroposterior, and mediolateral directions and the center of pressure were recorded using force platforms. Three typical gait instants as shown in Fig 2, the first-peak, mid-stance, and second-peak in terms of the vertical ground reaction force, were picked for simulation.

The gait pattern is thought to undergo some changes in cadence and stride length after ankle arthrodesis [4,9,14]. Few studies have reported the different boundary and loading conditions in terms of each specific instant. To make the ankle fusion the only independent variable in this study, the same boundary and loading conditions at corresponding instants were assumed in the ankle arthrodesis model.

The superior surfaces of the tibia, fibula, and encapsulated soft tissue were fixed throughout the simulation. The ground reaction forces in the vertical, anteroposterior, and mediolateral directions were applied through the rigid plate beneath the foot. The plate was also allowed to rotate for simulation of the foot shank angle (Fig 1). The degrees of freedom of translation in the vertical and anteroposterior directions and rotation along the anteroposterior direction were released, and the other three degrees of freedom were constrained. The muscle forces were initially estimated from the muscle cross-sectional area (PCSA) [54] and normalized electromyography (EMG) data during barefoot walking[55] with single muscle gain assumed for all muscles based on a linear EMG-force assumption [56]. The calculated muscle forces for this model were adjusted, based on another study of muscle activities in foot and ankle[57], to reasonably match the finite element predictions with captured motion events.

### Model validation

The finite element models were validated by comparison of the computational predictions with the experimental measurements. The plantar pressure in the balanced standing position and during walking calculated in the finite element model was compared with that obtained from the in vivo plantar pressure measurement. The contact pressure at the talonavicular joint predicted by the finite element method and measured using a cadaveric foot specimen was compared under the same boundary and loading conditions.

In the in vivo experiment, the plantar pressure distribution in the balanced standing position and during gait was measured using an F-Scan pressure measurement system (TekScan Inc., Boston, MA, USA). To obtain the measurement in the balanced standing position, the participant was asked to stand still on the F-Scan sensors for 5 seconds. The middle 3 seconds were selected and averaged. The plantar pressure during walking was collected from the gait experiment conducted for the boundary and loading conditions. The F-Scan sensor was cut to fit the foot size of the participant and attached to the plantar aspect of the right bare-foot using double faced adhesive tape. The plantar pressure was simultaneously collected and transmitted to the computer using wireless connection.

**Fig 3:**
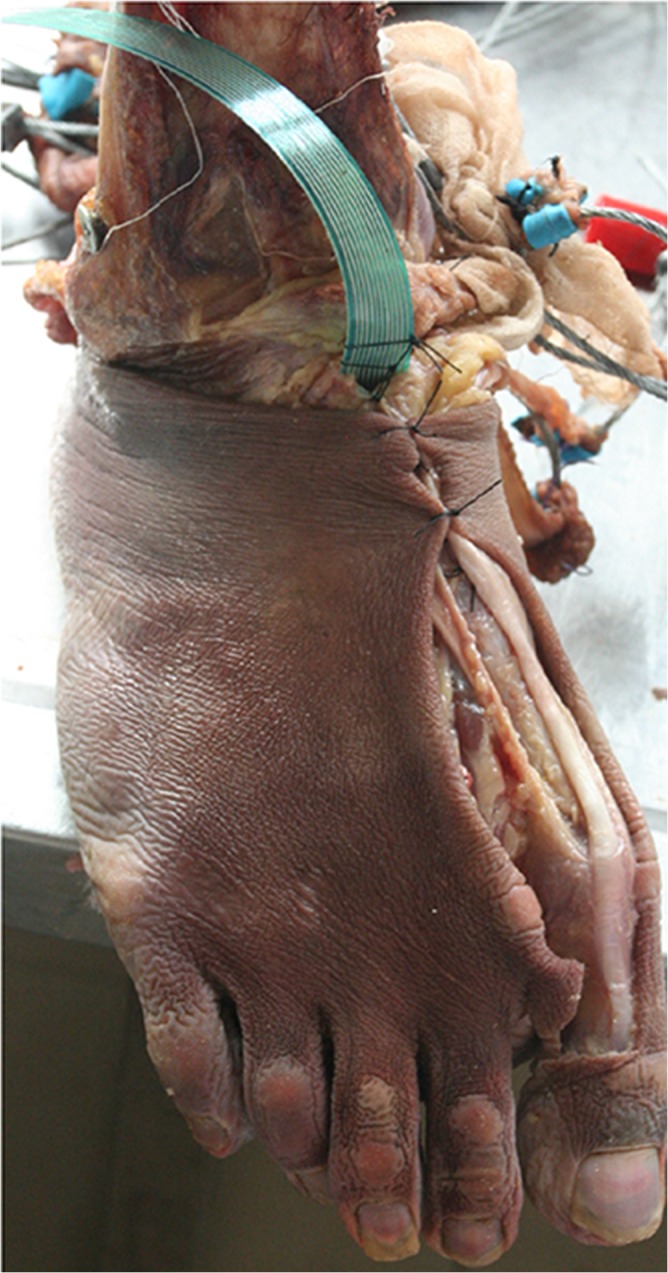
Cadaveric specimen of the foot and ankle.

A male cadaveric specimen consisting of part of the tibia and the entire foot sized of 42 was used in the cadaveric experiment. The contact pressure at the talonavicular joint was measured using a K-Scan sensor (TekScan Inc., Boston, MA, USA) under specific boundary and loading conditions implemented via a mechanical testing system (ElectroForce 3510, Bose, MT, USA). After the insertion of the K-Scan sensor from an incision on the dorsal aspect of the foot over the talonavicular joint (Fig 3), the tibia and fibula bones of the cadaveric foot were fixed on the testing machine in 10 degrees of dorsiflexion position through the adjustment of the rotational plate beneath the plantar foot. The tendons of extrinsic muscles were sutured to pulley lines for the convenience of muscle loading application. A compressive force of 100 N was applied vertically to the tibia and fibula cross-section, and muscle forces of 250 N for the Achilles tendon, 50 N for the tibialis posterior, 50 N for the flexor hallux longus, 50 N for the flexor digital longus, and 50 N for the peroneus longus were applied to the corresponding tendons. Simulations under the same boundary and loading conditions were executed in the finite element model.

## Results

### Model validation

**Fig 4:**
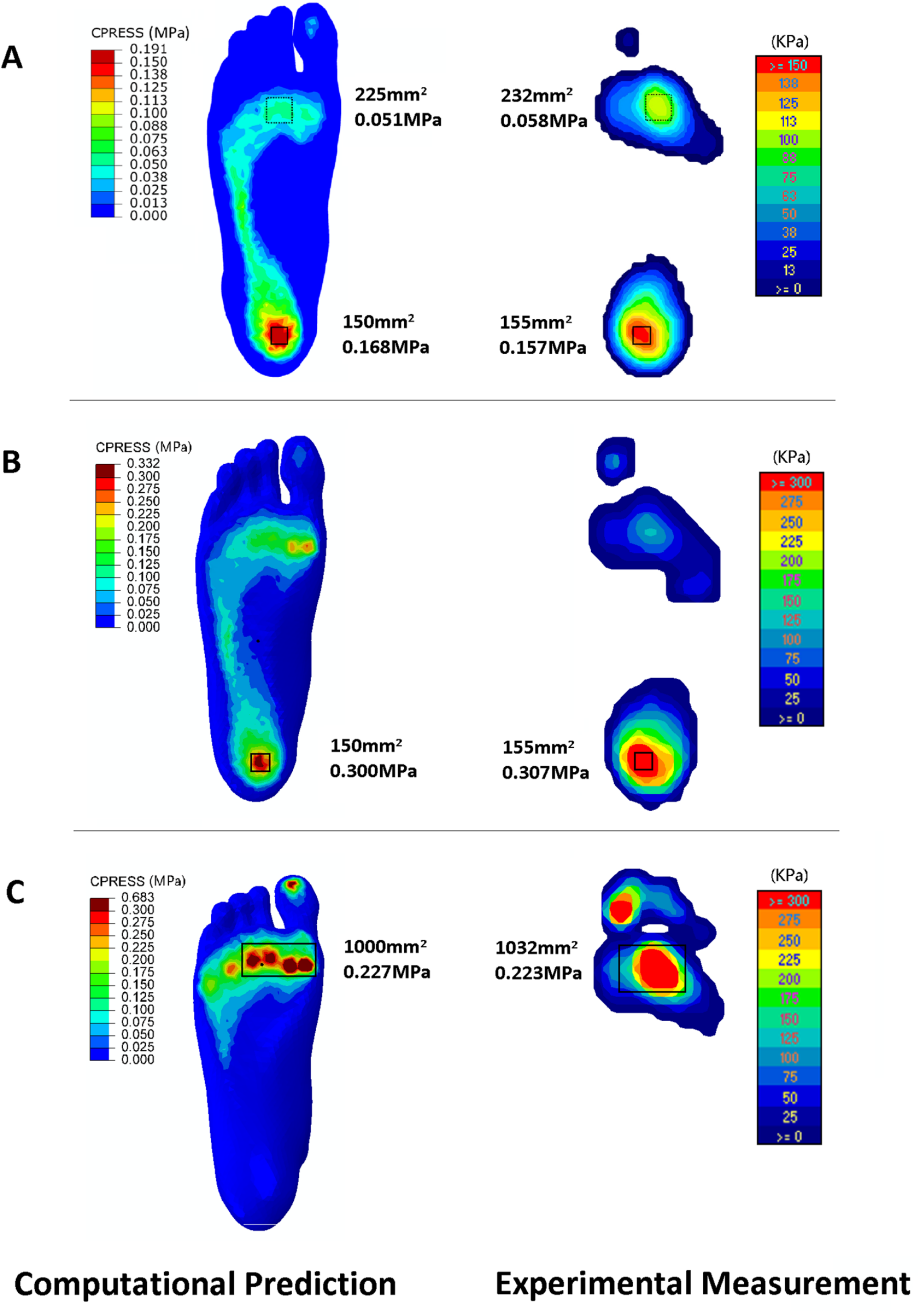
Comparison of the plantar pressure between computational prediction and experimental measurement in: A) balanced standing position, B) the first peak instant and C) the second peak instant for validation

Fig 4 shows comparisons of the plantar pressure distributions during the balanced standing and the first and second peak instants between the finite element prediction and the in vivo experimental measurements. To calculate the averaged pressure in concerned regions, rectangles or squares in equivalent areas were created in the model and F-Scan pressure map. The plantar pressure at each node in the involved elements was exported in the visualization module using the report menu and then these values were averaged in excel files. The same procedure was applied to the calculation of average joint contact pressure. The average pressure in selected areas of the F-Scan and K-Scan pressure maps could be directly obtained. In the balanced standing position, the peak pressure from both prediction and measurement was located beneath the heel. The average pressure over a small square area of 1.5 cm^2^ was 0.168 MPa and 0.157 MPa, respectively. The pressure beneath the heads of the second and third metatarsals was higher than in other regions over the fore-foot. The average pressure over a square area of 2.3 cm^2^ was 0.051 MPa and 0.058 MPa, respectively. At the first peak instant, the peak pressure was both located at the heel region and the average pressure over the 1.5 cm^2^ area was 0.300 MPa and 0.307 MPa respectively. The peak pressure at the second instants lied in the fore-foot region. The area of the first, second and third metatarsal heads sustained much higher pressure than other regions and thus the average pressure in a 10 cm^2^ area covering the first to third metatarsal heads was calculated. It was 0.227 and 0.223 MPa respectively in the finite element prediction and the F-scan measurement.

**Fig 5:**
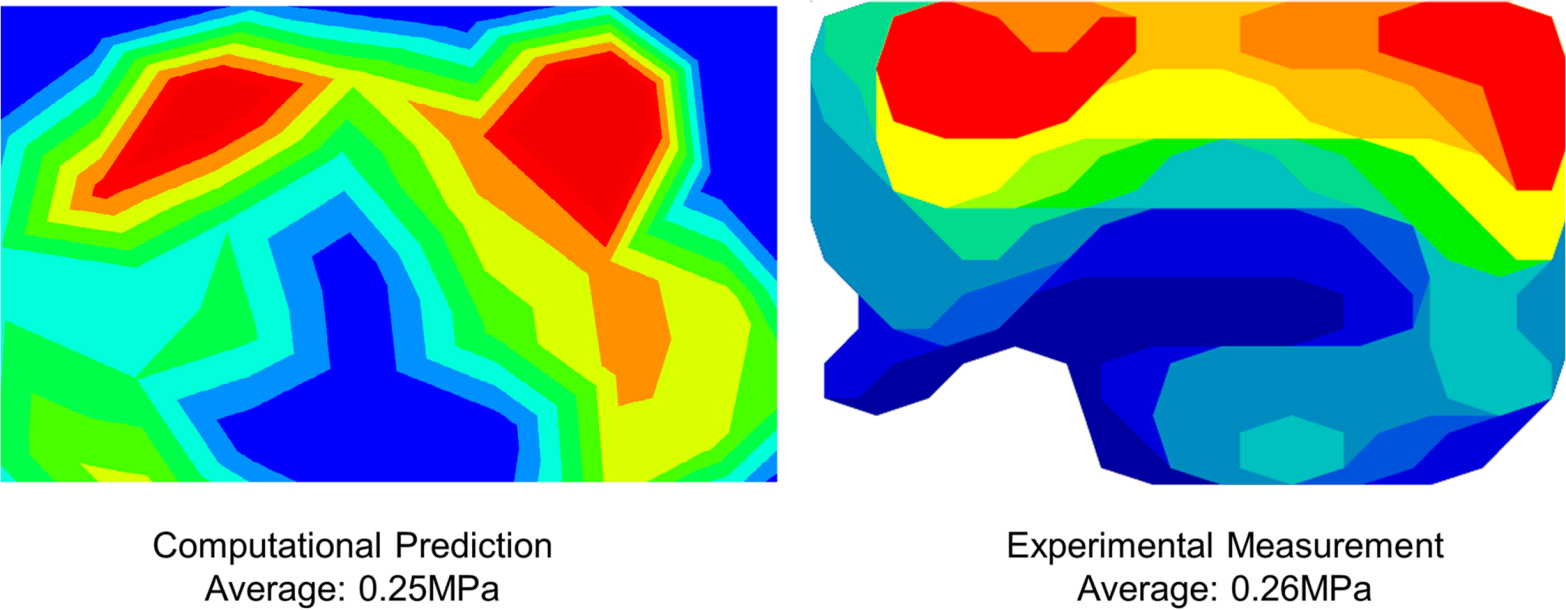
Comparison of the contact pressure at talonavicular joint between finite element prediction and cadaveric experiment measurement for validation.

Fig 5 shows a comparison of the contact pressure at the talonavicular joint interface under the same boundary and loading conditions from the finite element prediction and cadaveric measurement. Based on the size and location of the pressure sensor in the cadaveric measurement, an equivalent contact area was picked from the finite element model for comparison. The average pressure was 0.26 MPa and 0.25 MPa, respectively.

All comparisons show reasonable agreement between the finite element predictions and experimental measurements. The biomechanical performance of the foot and ankle was analyzed and compared using the validated model. The plantar pressure distribution, the contact pressure and force at eleven major joints (the subtalar joint, talonavicular joint, calcaneocuboid joint, three cuneonavicular joints, and five tarsometatarsal joints), and the stress in the five metatarsal bones were investigated.

### Plantar Pressure

**Fig 6:**
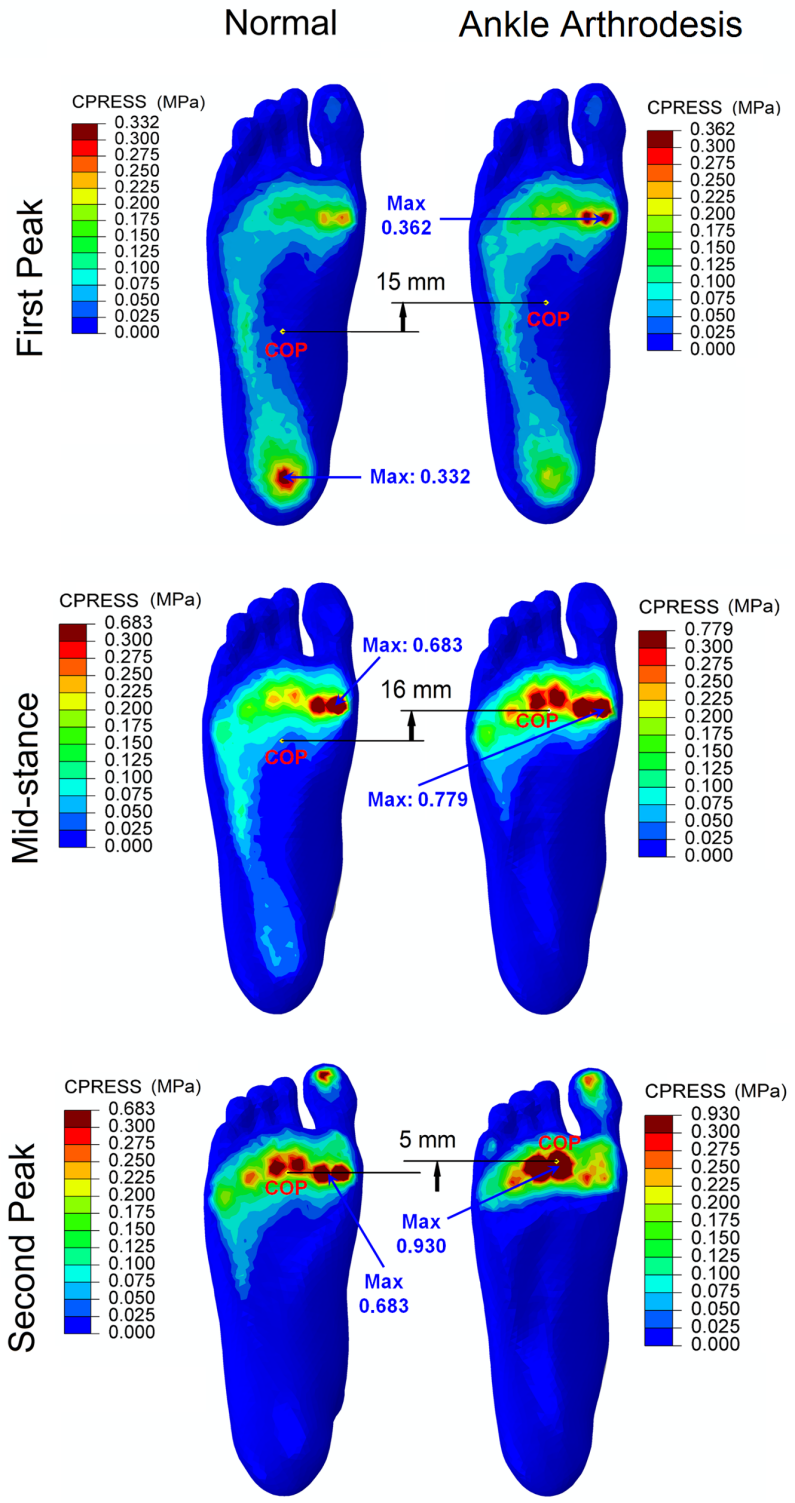
Comparison of the plantar pressure distribution between normal foot model and ankle arthrodesis foot model at the three instants.

Fig 6 displays the plantar pressure distributions at the first-peak, mid-stance, and second-peak instants in the normal and ankle arthrodesis models. The peak plantar pressure at the three respective instants was 0.33 MPa, 0.68 MPa, and 0.68 MPa in the normal model and 0.36 MPa, 0.78 MPa, and 0.93 MPa in the ankle arthrodesis model. In general, the peak plantar pressure increased due to ankle arthrodesis and was most obvious at the second-peak instant, especially over the fore-foot region. The center of pressure moved anteriorly by 15 mm, 16 mm, and 5 mm at the three instants. A slight variation in the center of pressure in the medio-lateral direction due to ankle arthrodesis was observed at the second-peak instant. It was located between the heads of the second and third metatarsal bones in the normal foot model and shifted medially to the head of the second metatarsal in the ankle arthrodesis model.

### Joint Contact Pressure

**Fig 7:**
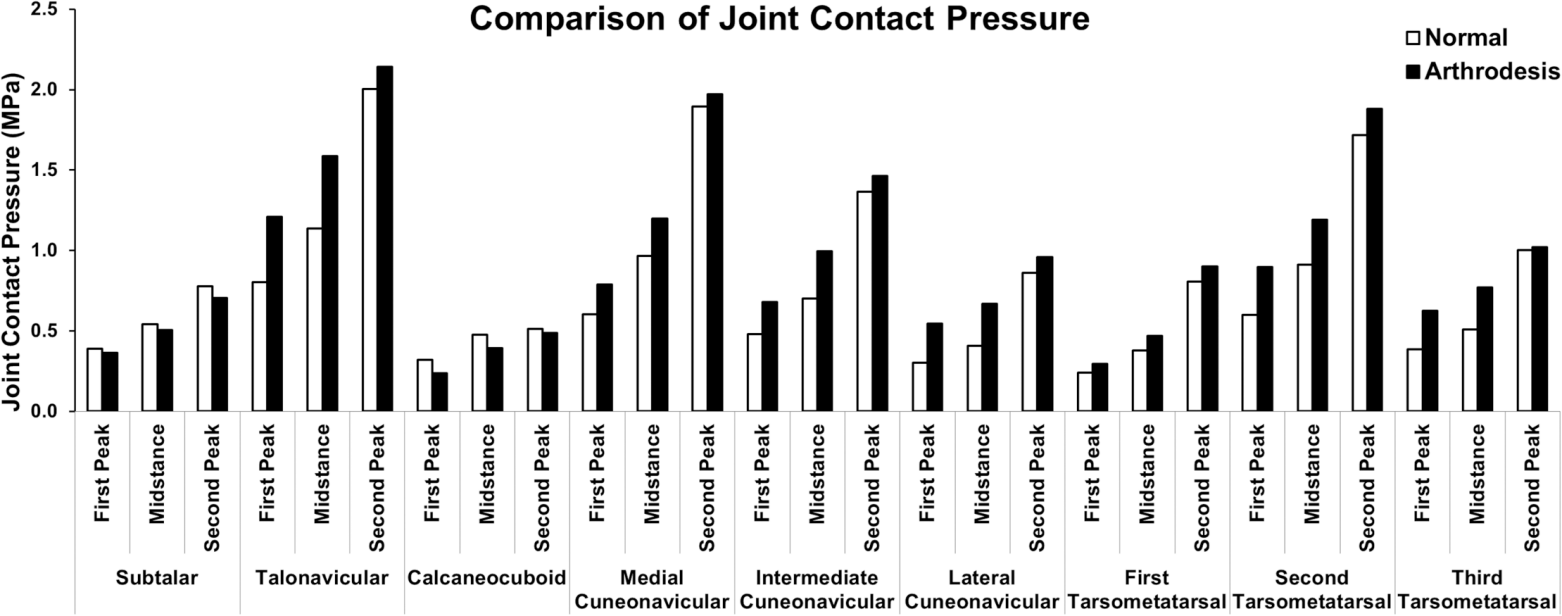
Comparison of the contact pressure at nine joints in the hind- and mid-foot between the normal foot model and the ankle arthrodesis foot model at the first-peak, mid-stance, and second-peak instants.

Fig 7 shows the contact pressure at nine major joints at the three gait instants of the normal and ankle arthrodesis models. The contact pressure at the talonavicular and intertarsal joints, including the three cuneonavicular joints and the first three tarsometatarsal joints, was larger in the ankle arthrodesis model than in the normal foot model. The average contact pressure at the talonavicular joint was the highest among all of the analyzed joints. The ankle arthrodesis caused the contact pressure to increase from 0.80 MPa, 1.14 MPa, and 2.00 MPa to 1.21 MPa, 1.59 MPa, and 2.14 MPa at the first-peak, mid-stance, and second-peak instants, respectively.

Among the three cuneonavicular joints, the medial joint underwent greater contact pressure than the other two. The respective pressure was 0.60 MPa, 0.97 MPa, and 1.90 MPa in the normal foot and 0.79 MPa, 1.20 MPa, and 1.97 MPa in the ankle arthrodesis foot at the three gait instants. The maximum variation due to ankle arthrodesis occurred at the lateral cuneonavicular joint and was 80.3%, 64.7%, and 11.6% at the first-peak, mid-stance and second-peak instants, respectively.

Among the three tarsometatarsal joints, the second tarsometatarsal joint underwent the highest contact pressure, which was 0.60 MPa, 0.91 MPa, and 1.72 MPa in the normal foot and 0.90 MPa, 1.19 MPa, and 1.88 MPa in the ankle arthrodesis foot, at the three gait instants. The ankle arthrodesis resulted in the biggest variation at the third tarsometatarsal joint. The variation was 62.8%, 51.4%, and 2.0% at the three instants, respectively. The contact pressure at the fourth tarsometatarsal joint increased by 63.1% at the second-peak instant and decreased at the other two instants due to ankle arthrodesis. The contact pressure at the fifth tarsometatarsal joint increased at the first-peak instant and decreased at the other two instants.

Reduced contact pressure due to ankle arthrodesis was found at the subtalar and calcaneocuboid joints. The respective contact pressure at the three instants at the subtalar joint was 0.39 MPa, 0.54 MPa, and 0.78 MPa in the normal foot and 0.37 MPa, 0.51 MPa, and 0.70 MPa in the ankle arthrodesis foot, respectively. The contact pressure at the calcaneocuboid joint was 0.32 MPa, 0.48 MPa, and 0.51 MPa in the normal foot and 0.24 MPa, 0.39 MPa, and 0.49 MPa in the ankle arthrodesis foot.

### Joint Contact Force

**Fig 8:**
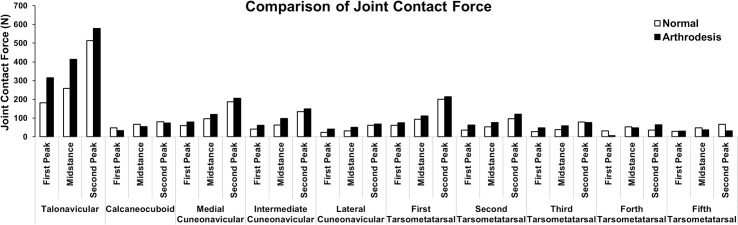
Comparison of the contact forces at ten joints in the hind- and mid-foot between the normal foot model and ankle arthrodesis model at the first-peak, mid-stance, and second-peak instants.

Fig 8 depicts the normal contact forces at the 10 joints at the three gait instants in the normal and ankle arthrodesis models. The contact force at the talonavicular joint, the three cuneonavicular joints, and the first and second tarsometatarsal joints increased at the three instants due to ankle arthrodesis. For most of the joints, the maximum magnitude of the contact force occurred at the second-peak instant, whereas the maximum variation occurred at the first-peak instant.

The talonavicular joint undertook the greatest force among all of the joints. The force was 181 N, 259 N, and 514 N at the three instants, and increased by 74%, 60%, and 12% in the ankle arthrodesis model. The maximum contact force at this joint occurred in the ankle arthrodesis model at the second-peak instant and was 578 N, slightly larger than the body weight of 540 N. In contrast, the contact force at the other transverse tarsal (calcaneocuboid) joint decreased by 31%, 17%, and 8%, with a force of 33 N, 55 N, and 74 N at the three gait instants due to ankle arthrodesis.

Joints of the first ray (medial cuneonavicular and first tarsometatarsal joints) withstood higher a contact force than those of the second ray (intermediate cuneonavicular and second tarsometatarsal joints) and third ray (lateral cuneonavicular and third tarsometatarsal joints). Joints in the first three rays experienced a greater contact force at the second-peak instant than the first-peak and mid-stance instants in both models. The maximum variation occurred at the first-peak instant. The contact forces at the two joints of the first ray increased by 31% and 75% at this instant whereas it increased in the second ray joints by 52% and 79%, and was predicted to increase in the third ray joints by 74% and 71%.

**Fig 9:**
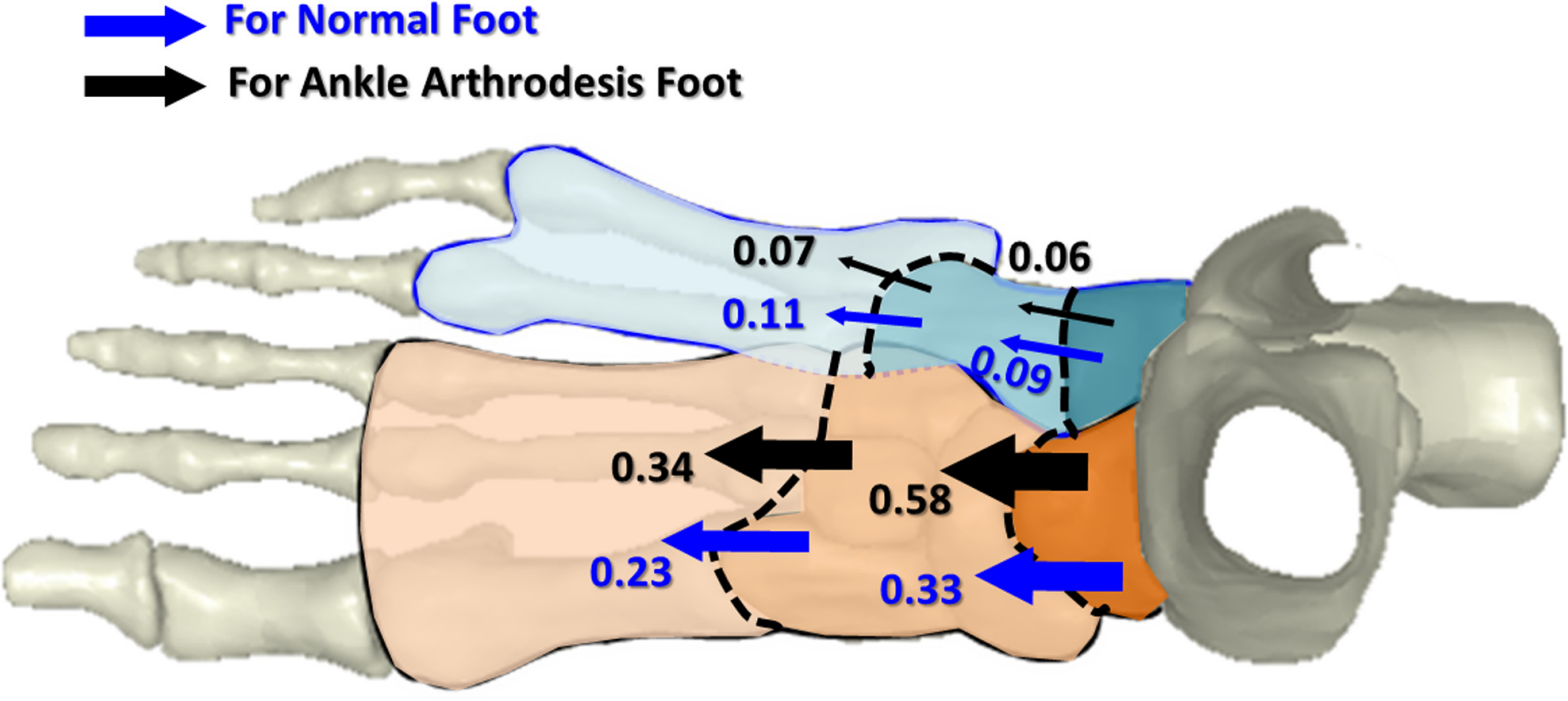
Load transfer (times of body weight) in the normal and ankle arthrodesis foot model at the first-peak instant.

Fig 9 shows the variation in the load transfer distribution between the normal and arthrodesis models at the first-peak instant, at which the arthrodesis effect is most obvious compared with the other two instants. In the normal foot, about 0.33 times body weight was transferred through the talonavicular joint to the first three rays and about 0.09 times body weight through the calcaneocuboid joint to the fourth and fifth rays. The mid-foot transferred 0.23 times weight body to the fore-foot through the first three rays and 0.11 through the fourth and fifth rays. In the ankle arthrodesis foot, the load transferred through the talonavicular joint increased to 0.58 times body weight and decreased to 0.06 at the calcaneocuboid joint. The force transferred from mid-foot to fore-foot increased to 0.34 times body weight in the first three rays and decreased to 0.07 in the two lateral rays. In general, the transfer of force shifted to the medial side due to ankle arthrodesis.

### Bone Stress

**Fig 10:**
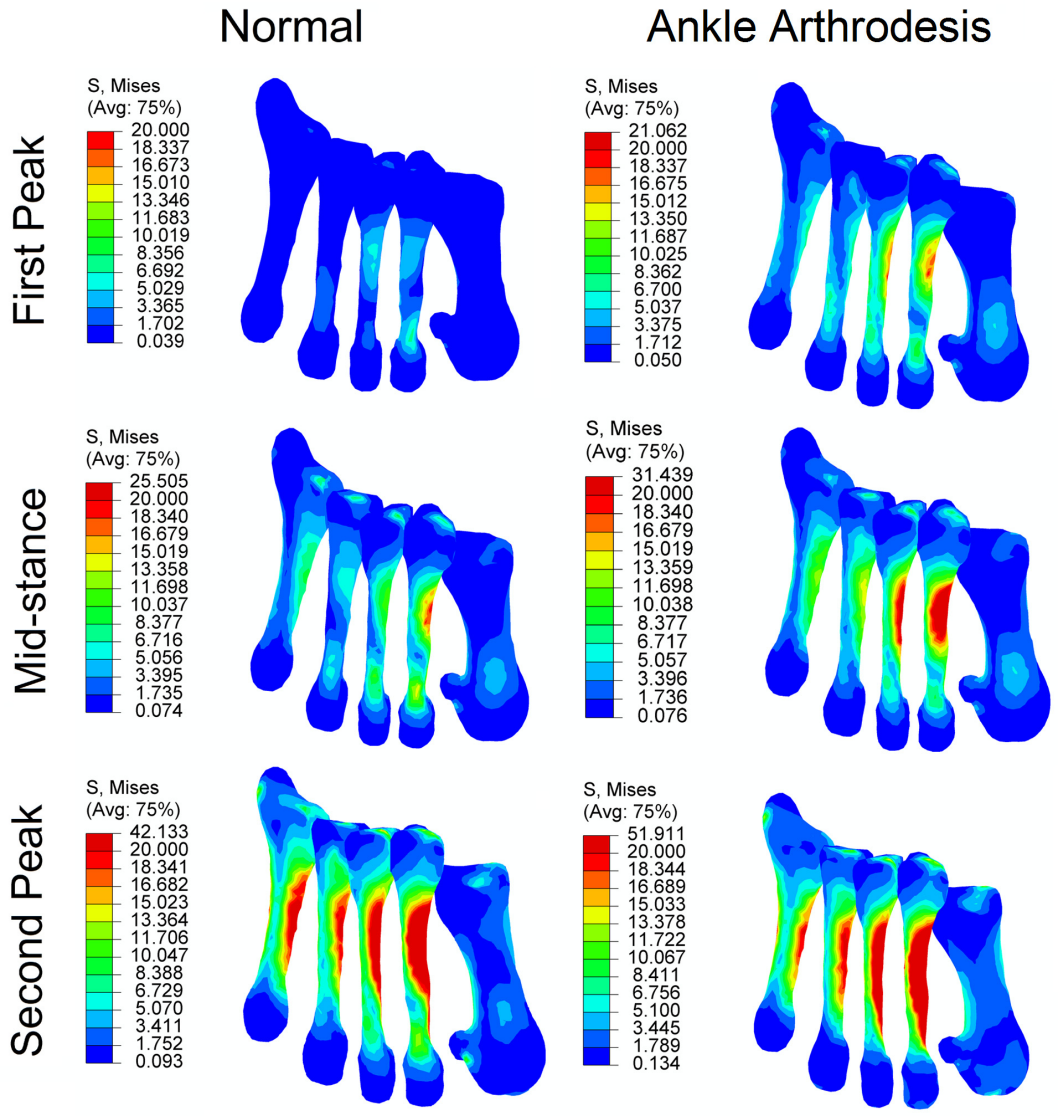
Comparison of von Mises stress in five metatarsal bones in normal foot model and ankle arthrodesis foot model at three instants.

The von Mises stress in bone generally increased due to the ankle arthrodesis, as shown in Fig 10. At the first-peak instant, the von Mises stress in the first and third metatarsal bones increased by 19% and 52%, respectively, due to ankle arthrodesis, whereas the stress in the other three metatarsals remained unchanged or slightly decreased. At the mid-stance and second-peak instants, stress in all five metatarsal bones showed an obvious increase. The maximum stress was found in the second metatarsal bone at the second-peak instant among all of the simulations. It was 42 MPa in the normal foot and increased to 52 MPa due to ankle arthrodesis. The stress in the third metatarsal bone was 20 MPa at mid-stance and 34 MPa at the second-peak instant in the normal foot and increased by 39% and 20%, respectively, in the ankle arthrodesis foot. The second and third metatarsals bore much greater stress than the other three metatarsals.

### Foot deformation at the second-peak instant

**Fig 11:**
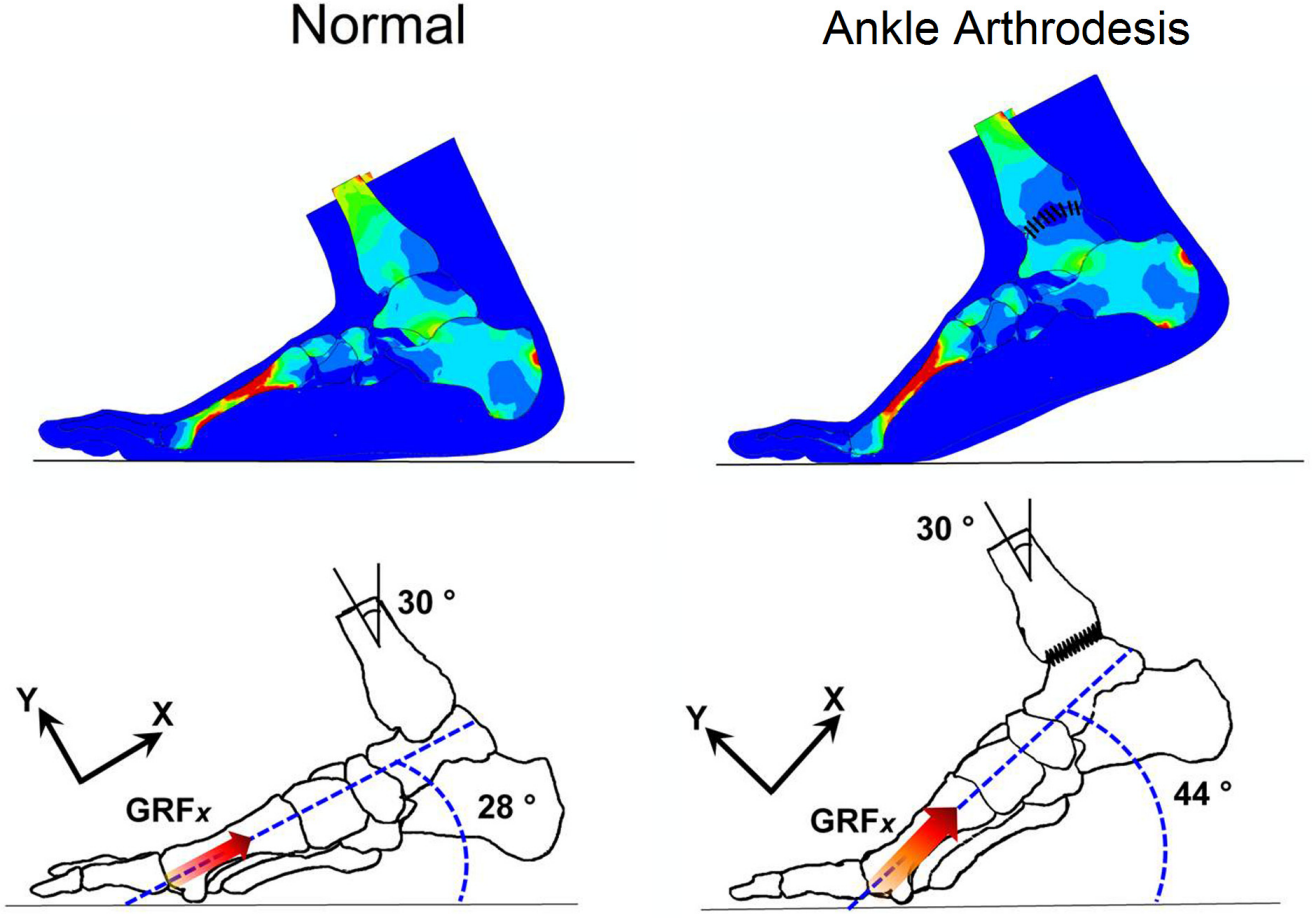
Angular positions of normal foot and ankle arthrodesis foot at second-peak instant in sagittal plane.

The finite element analysis showed different foot deformations between the ankle arthrodesis and normal foot. The angular displacement of the hind- and mid-foot, especially at the second-peak instant, clearly changed. As illustrated in Fig 11, the angle between the ground and the axis along the first ray was 28° in the normal foot and 44° in the ankle arthrodesis foot at the second-peak instant. At the same boundary condition, the foot shank angle was 30° from the vertical direction.

## Discussion

In this study, three-dimensional comprehensive finite element models of the foot and ankle involving the major anatomical structures were developed. This model was validated using comparison of two parameters between experimental measurements and model predictions, including plantar pressure during balanced standing and walking and joint contact pressure under specific boundary and loading conditions. Biomechanical performances were simulated in both a normal foot model and an ankle arthrodesis model. The boundary and loading conditions were obtained from motion analysis study. The detailed representation of the anatomical structures and measured boundary and loading conditions make the model capable of predicting with reliable insight the biomechanical behavior of each individual segment and the entire foot variation with any type of surgical treatment. As the model in this study, the latest finite element models of foot and ankle were mainly reconstructed from computed tomography or magnetic resonance images, which could accurately replicate anatomical contours of segments. Some of these models consisted of the majority anatomical structures of the entire foot and ankle complex[34,39,58–63], while others were developed into specific parts[32,64,65]. The tissue properties were mostly assigned as linear or non-linear elasticity and viscoelasticity has been taken into account for dynamic simulations. The validation of models have been demonstrated in some of these studies and the methods could be comparison between finite element prediction and physical measurements[33,36,38,61,63], and/or data from literatures[32,61,62].

The foot and ankle complex is a synergetic system and its individual segments interact interdependently. Constrained ankle motion resulted in substantial variations in the biomechanical behavior of the entire foot, as predicted. The angular displacement between the fore- and mid-foot was much larger in the ankle arthrodesis foot than in the normal foot, which may be compensation for the constrained ankle motion. These kinematic changes result in abnormal load transfer among foot segments, reflected in the changes in plantar pressure, joint contact pressure and force, and bone stress distribution. These variations may provide indications for outcome assessment of ankle arthrodesis surgery.

Regarding plantar pressure distribution, the location and magnitude of peak pressure clearly changed in the ankle arthrodesis model compared with the normal model. At the first-peak instant, the location of the peak pressure changed from beneath the heel to the fore-foot beneath the head of the first metatarsal bone. At the first-peak instant at 27% of the stance phase, the foot was dorsiflexed by about 6 degrees, as recorded in the gait analysis of this and previous studies [15,55,66]. The foot dorsiflexion and the forward tilt of the tibia bone during gait progression were compensated for by the increased displacement of the bones between the metatarsal heads and ankle joints [4,12,66,67] in the sagittal plane, which is consistent with our prediction. This compensation could have driven the earlier heel-off [12] and the greater anterior center of pressure than that in the normal foot. A greater anterior center of pressure induces a longer moment arm of ground reaction force to the hind- and mid-foot joints and is presumably a contributor to the deterioration in the loading of the foot, as predicted in this study. The increase in plantar pressure could be a risk factor for foot pain after surgery. These secondary impairments can result in discomfort during gait. Patients tend to adjust their gait pattern in terms of walking speed and cadence [68] to minimize these effects.

The talonavicular joint undertook a maximum contact pressure of up to 2.14 MPa at the second-peak instant after ankle arthrodesis. As earlier demonstrated, excessive contact stress at the articular surface is believed to be a predominant factor of osteoarthritis [69]. These variations could be regarded as a predictor of arthritis at mid-foot joints, including the talonavicular, cuneonavicular, and tarsometatarsal articulations, as reported in retrospective studies of negative outcomes of ankle arthrodesis [6,8,51,70].

Ipsilateral hind-foot arthritis, particularly in the subtalar joint, has been shown to be an outcome of ankle arthrodesis in retrospective studies and is thought to be induced from increased loading in the ankle arthrodesis foot [4,6,51,71]. In this study, however, the contact pressure and transferred force at the subtalar joint were decreased in the arthrodesis foot at the three gait instants. This provides a basis to speculate that subtalar arthritis may not be a consequence of ankle arthrodesis, but rather a progression of pre-existing degenerative changes at this joint, which are demonstrated universally in patients requiring arthrodesis. A study of the relationship between hind- and mid-foot arthritis and ankle arthrodesis found that 68 of 70 patients showed pre-existing arthritis, mostly in the subtalar joints [8]. The hypermobility of the subtalar joint accentuates rotatory moments [72]. The talus bone rotates 4.5 degrees during foot dorsi- and plantarflexion in the normal foot [22]. After ankle arthrodesis, the movement of the talus is constrained to the tibia bone and results in decreased mobility of the subtalar joint. It can be speculated that the decreased mobility of the subtalar may release part of the load acting at this articulation.

The load is transmitted from the hind-foot to the mid-foot through the transverse tarsal joints consisting of the talonavicular and calcaneocuboid articulations. The talonavicular joint bears the major force [73] and delivers it to the first three rays, at about 78.6% in the normal foot and 90.6% in the ankle arthrodesis foot at the first-peak instant, as shown in Fig 9. Variation in the load transfer path may result in foot pain in the medial columns, either in the soft tissue or bones, and a long-term outcome of foot deformity. Orthotic treatments such as wedged insoles may help to release part of these effects after surgery.

The increased force in the first three rays induced increased stress in the first three metatarsal bones. As predicted, the second and third metatarsal bones sustained much higher von Mises stress than the other metatarsal bones. As they have relatively thinner and longer geometries than other foot bones and have a loading transfer function, the metatarsal bones are thought to be most susceptible to bone fractures. It was reported that the second and third metatarsal bones most commonly suffer stress fractures, and fracture of the second metatarsal is one of the most common complaints after foot and ankle surgery [74]. The predictions in this study may explain this clinical phenomenon. In both the normal and ankle arthrodesis foot, the two metatarsals bore much higher stress than other bones. Ankle arthrodesis induced the most stress increase in these two bones. As the von Mises stress is considered to be the predictor for bony stress fracture [75], it can be speculated that patients with ankle arthrodesis are more susceptible to stress fractures in the second or third metatarsal bones.

There is a risk of volumetric locking when using 4-noded tetrahedral formulations for modeling of incompressible/nearly incompressible continua [76–78]. Reduced integration can be adopted to avoid locking, and the cartilaginous structures assigned with Poisson’s ratio of 0.4 were meshed using 4-node linear tetrahedron elements instead of quadratic tetrahedron to reduce integration points in this study.

The balanced standing is one of the most common behaviors and promises a stable measurement of plantar pressure and is preferred in calibration of many motion analysis and physical examinations. The plantar pressure in this position was measured and adopted for the validation of the finite element model in this study. The first and second peak instants are two characteristic points at which the vertical ground reaction force from the force platform and the contact force from the F-scan sensor were well fitted. The plantar pressure at these two instants was measured simultaneously during the gait analysis and was compared to the corresponding finite element predictions. The talonavicular joint occupies relatively large contact area and functions as the major path of force transmit between the hind- and mid-foot. Moreover, it was found to be well fit the sensor in our experiment due to the contour of the joint interface and the large contact area. Thus, the contact pressure at this joint was selected as another parameter for validation. To reduce the effect of the foot size difference between the model subject and the cadaveric specimen, which could result in some variation of contact area of joints, relatively smaller area covering the two higher contact pressure points was selected at the talonavicular joint in the model to compare with the corresponding K-Scan measurement. Beside of the two instants with higher contact pressure in the two pressure maps, the area at the middle and left side of the bottom area sustained relatively smaller or little pressure. In the K-Scan sensor there was no contact data recorded at the left bottom corner while the computational prediction shows the pressure in this area. This might be due to the deviation of the contour of the articular interface in the model from reality. Due to the identifiability of the magnetic resonance images and the threshold value determined in MIMICS, the contour of the articular interfaces could not be precisely represented in the model. Averaged pressures in concerned area were analyzed for comparison to reduce the effect of the density difference between element in the model and measuring unit in the sensors.

## Limitations

The computational models in this study were based on some simplifications and assumptions. The bones of the finite element model of the foot and ankle were reconstructed without separation of cortical and trabecular components and assigned as a homogeneous, isotropic, and linear elastic material. The property constants, Young’s modulus of 7300 MPa and Poisson’s ratio of 0.3 were originally assumed[79] without experimental support and was defended to be a weighted average of cortical and trabecular elasticity properties based on their volumetric contribution[44]. Further experimental study should be conducted for a more objective evaluation of the bone property and the reasonability of the current assumptions. Due to the simplification of the bone components and assumption of the bone property the stress distribution in metatarsal bones were expected to demonstrate the variation trend of the force transmission resulted from ankle arthrodesis, rather than an exact representation of real cases. To explore the load transfer mechanism through comparison of other exclusive independent factors rather than ankle motion, the foot with ankle arthrodesis was simulated under the same gait pattern as the normal foot. There are claims that arthrodesis does not change the gait pattern, but some patients may adjust both the weight-bearing between their two feet and the duration of the stance phase. The ankle arthrodesis was simulated by tying the contact pair of the ankle articulation rather than using screws or pins. Although the concerned performance in this study was out of the ankle joint area, it would be better to reconstruct the screws for further investigation of the ankle joint behavior and the feasibility of the screws. The ankle is fused in a neutral position based on clinical recommendations. However, this is not universally adaptable to all cases because many different protocols have been adopted, such as slight dorsiflexion and 5 to 10 degrees of heel valgus. Three featured gait instants were simulated in this study. To represent the gait activity more closely, it will be necessary to simulate more instants in a further study.

## Conclusions

Ankle arthrodesis is used to relieve pain and improve the function of a foot with ankle degeneration. However, it alters the biomechanical performance of the foot and ankle, and some alterations may result in postoperative complications. These alterations occur not only around the operative sites but also in the entire foot and ankle.

Changes in biomechanical parameters, such as the plantar pressure distribution, joint contact pressure and forces, von Mises stress in bones, and foot deformation, are possible indications of the consequences of ankle arthrodesis. They may be used to explain some clinical observations. Large alterations at the talonavicular joint and the joints of the first three rays may give rise to pain and the potential for arthritis. Large stress exerted in the second and third metatarsal bones may cause bone fractures. The decrease in subtalar joint loading after ankle arthrodesis may indicate that the postoperative arthritis of the subtalar joint is not be a consequence of ankle arthrodesis but rather a progression of pre-existing joint degeneration.

The information on the inner foot provided in this study can serve as a baseline for the optimization of surgical protocols and interventions for rehabilitation. A slight valgus foot position for ankle arthrodesis may distribute more loading from the first three rays to the lateral rays of the foot to prevent adverse changes. In addition to adjustments to the surgical procedure, orthotics [9,70] such as insoles or canes can help to relieve the detrimental loading effects. Further evaluation studies are needed to verify the effectiveness of these interventions.
